# Implications of gender-based variabilities in bone mineral density and hemoglobin levels

**DOI:** 10.1186/s12891-021-04536-7

**Published:** 2021-07-30

**Authors:** Yumei Zhou, Shengjun Liu, Ximei Wang, Yuhan Fu, Fan Su, Lei Cao, Xiaojuan Zha, Yufeng Wen

**Affiliations:** 1 School of Public Health, Wannan Medical College, 22 West Wenchang Road, Wuhu, Anhui Province 241002 People’s Republic of China; 2 Institute of Quantitative Pharmacology Department of Pharmacology, Wannan Medical College, 22 West Wenchang Road, Wuhu, Anhui Province 241002 People’s Republic of China; 3 First Affiliated Hospital, Wannan Medical College, 2 West Zheshan Road, Wuhu, Anhui Province 241004 People’s Republic of China; 4 School of Laboratory Medicine, Wannan Medical College, 22 West Wenchang Road, Wuhu, Anhui Province 241002 People’s Republic of China

**Keywords:** Volumetric bone mineral density, Hemoglobin, Genders, U-shaped curve, Linear relationship

## Abstract

**Background:**

Studies reported that there is a relationship between volumetric bone mineral density (vBMD) and hemoglobin (HGB) in sickle cell anemia, chronic obstructive pulmonary disease, inflammatory bowel disease, and chronic kidney disease, it is not clear whether this association exists in normal populations or different genders. In order to further clarify the relationship between vBMD and HGB, and provide the basis for the diagnosis of related diseases, this study was conducted in the physical examination population.

**Methods:**

A cross-sectional study was conducted on a health check-up population from Wannan area of China from January to December 2018. The study involved 1238 individuals aged 23 to 85 years. Linear regression analysis and smooth curve were applied to determine the relationship of HGB and vBMD.

**Results:**

The average level of vBMD in the population was 130.11 ± 79.51 mg/cm^3^, after adjusting for age, body mass index (BMI), systolic blood pressure (SBP), diastolic blood pressure (DBP), total cholesterol (TC), triglycerides (TG), glucose (GLU), high-density lipoprotein (HDL) and low-density lipoprotein (LDL). A U-shape relationship was established between vBMD and HGB, the cut off value of HGB was 130 g/L. After gender stratification, the results showed a U-shaped curve relationship between vBMD and HGB in male group, and a linear relationship between vBMD and HGB in female group. The vBMD decreased with HGB when HGB < 120 g/L, and increased when HGB ≥ 120 g/L in male group.

**Conclusion:**

The relationship between vBMD and HGB in the male physical examination population presents a U-shaped curve.

**Supplementary Information:**

The online version contains supplementary material available at 10.1186/s12891-021-04536-7.

## Background

Osteoporosis (OP) is a systemic bone metabolic disease characterized by decreased bone mass, increased bone fragility, and susceptibility to fractures [1]. There are approximately 200 million osteoporotic patients worldwide, and about 8.9 million fractures occur every year globally [2–4]. Previous studies have identified the basic metabolic conditions and risk factors associated with osteoporosis, these factors include age, weight, dyslipidemia and hypertension [5] and so on. The most common diagnostic criterion for OP is bone mineral density (BMD) [2]. The strategies of treating osteoporosis are exercise and pharmacological treatments which involves drugs such as risedronate [6], alendronate [7], estrogens [8, 9], SERMs [10–12] and bisphosphonates [13–16]. However, there is no treatment method that is fast and effective enough to deal with OP [17, 18]. Thus, OP is still a major public health problem [19].

Studies have reported a positive correlation between BMD and hemoglobin (HGB) levels in patients with sickle cell anemia, chronic obstructive pulmonary disease, inflammatory bowel disease, and chronic kidney disease [20–23], while some other studies found that there was no significant correlation between BMD and HGB [24–26]. These two indicators correspond to the diagnosis of different systemic diseases, in addition to focusing on the correlation between them, other aspects of the research are limited. Whether there is a nonlinear relationship between them or does this correlation exist in normal people is not clear. Therefore, further clarify the relationship between them is still very necessary. This cross-sectional study is to investigate the relationship between volumetric bone mineral density (vBMD) and HGB in a relatively wide age range health check-up population.

## Materials and methods

### Study subjects

A cross-sectional study was conducted on 1238 health check-up participants aged 23 to 85 years in the Health Management Center at the First Affiliated Hospital of Wannan Medical College in Wuhu, China from January to December 2018. All protocols are carried out in accordance with relevant guidelines and regulations. Ethical approval was received from the Medical Ethics Committee of Wannan Medical College. Since the participants in this study are the people who came to the hospital for routine physical examinations, the Medical Ethics Committee of Wannan Medical College approved that the study only needs to obtain the participant’s verbal informed consent. Therefore, this study did not have the participants’ written informed consent. Before the survey, each participant provided verbal informed consent. In addition, data related to private information were deleted during analysis of the results. The participants were: (1) subjects within the age range of 20—90 years, and (2) subjects with available data on age, sex, HGB, SBP, DBP, TG, TC, GLU, low-density lipoprotein (LDL), high-density lipoprotein (HDL), and vBMD. All subjects completed physical examination and blood biochemical examination. The following categories of subjects were excluded from the study: (1) subjects with incomplete clinical data, duplicated cases, and missing data; (2) subjects who received drug interventions, and (3) patients who had cancer, severe cardiovascular disease or severe infections. The study involved a total of 1238 participants made up of 525 females (42.41%) and 713 males (57.59%). The mean ages of the female and male participants were 53.07 ± 12.94 and 52.38 ± 12.86 years, respectively.

### Study questionnaire

A questionnaire was used to collect information with respect to demographic and behavioral characteristics, disease history and surgical history [27]. The demographic characteristics included age, sex, occupation and educational background, while the behavioral characteristics included smoking and drinking. The other information obtained were disease history, surgical history and history of severe infections.

### Physical examination

Trained professionals measured height, weight, blood pressure and other variables using standard methods, based on the guidelines of World Health Organization (WHO) and International Society of Hypertension [28]. Height measurement required subjects to stand barefooted on the floor of the height meter, with their trunks naturally straight, heads straight, with eyes focused in front, upper limbs drooping naturally, and legs straight. The recorded data on height were accurate to 0.1 cm. Body weight measurement required barefooted subjects to wear shorts and stand naturally in the center of the weight pedal so as to keep their bodies stable. The recorded data were accurate to 0.1 kg. The BMI was calculated by dividing body weight (kg) with the square of the height (m) i.e., m^2^, to an accuracy of 0.01 kg/m^2^. Systolic blood pressure (SBP) and diastolic blood pressure (DBP) was measured with a mercury sphygmomanometer. The subjects were advised to sit still for 5 min before the BP measurement.

### Blood parameters

Fasting venous blood (5 mL) was collected from each subject in the morning, and all samples were analyzed within 24 h. The indicators included TG, TC, GLU, LDL and HDL were measured using the Hitachi 7600 automatic biochemical analyzer.

### Bone mineral density examination

The lumbar spine was scanned with quantitative CT (QCT) method [29]. The subject lay supine on a CT scanning bed, and a standard phantom and blue cushion was placed under the lumbar spine parallel to the body. The blue soft cushion was placed close to the lumbar spine without any gap during the period. The value of vBMD was defined as the mean value of vBMD of the lumbar spine from L1 to L4.

### Statistical analyses

Statistical analyses were performed using SPSS 18.0 software and R-project 2.41. The continuous variables are all normal distribution and presented as mean ± standard deviation. Student’s *t*-test was used to compare the differences of demographics characteristic, and indicators of physical examination and blood biochemical examination between men and women. The relationship between vBMD and HGB was tested using generalized smoothing splines, and the knot locations was generated automatically in generalized additive models with R package MGCV [30]. Adjusted factors were age, BMI, GLU, TC, TG, SBP, DBP, HDL and LDL. According to the knot locations of HGB, the relationship between HGB and vBMD in different HGB segments was further analyzed with linear regression model, and adjusted age, BMI, GLU, TC, TG, SBP, DBP, HDL and LDL. On this basis, gender was stratified, and the relationship between HGB and vBMD was analyzed with generalized smoothing splines, then the linear regression was conducted to analyze the relationship between HGB and vBMD in different HGB segments according to the knot locations in different genders, and adjusted age, BMI, GLU, TC, TG, SBP, DBP, HDL and LDL. All *p* values were two-tailed, with a significance level of 0.05.

## Results

### General characteristics of male and female participants

Table 1 shows that HGB, BMI, SBP, DBP, TC, TG, GLU, HDL and LDL differed significantly between the male and female groups (*p* < 0.05).

**Table 1:** Comparison of demographic characteristics and biochemical indicators by genders

Variable	Male	Female	*t*	*P*
vBMD (mg/cm^3^)	130.99 ± 34.81 (38.14—247.94)	129.69 ± 46.11 (22.25—258.25)	0.57	0.569
HGB (g/L)	148.33 ± 11.15 (95—183)	127.88 ± 12.16 (52—157)	31.03	0.000
AGE (years)	52.38 ± 12.86	53.07 ± 12.94	-0.92	0.358
BMI (kg/m^2^)	24.81 ± 2.99	23.22 ± 3.12	9.04	0.000
SBP (mm Hg)	134.70 ± 16.26	132.06 ± 20.37	2.53	0.012
DBP (mm Hg)	81.67 ± 10.06	76.71 ± 9.86	8.62	0.000
TC (mmol/L)	4.58 ± 0.92	4.70 ± 0.87	3.015	0.003
TG (mmol/L)	1.96 ± 1.48	1.48 ± 0.91	6.64	0.000
GLU (mmol/L)	5.37 ± 1.31	5.10 ± 0.98	3.91	0.000
HDL (mmol/L)	1.29 ± 0.28	1.52 ± 0.31	-13.36	0.000
LDL (mmol/L)	2.40 ± 0.79	2.51 ± 0.73	-2.48	0.013

*vBMD* volumetric bone mineral density, *HGB* hemoglobin, *BMI* body mass index, *SBP* systolic blood pressure, *DBP* diastolic blood pressure, *TC* total cholesterol, *TG* triglycerides, *GLU* glucose, *HDL* high-density lipoprotein, *LDL* low-density lipoprotein

### Relationship between vBMD and HGB

**Fig. 1:**
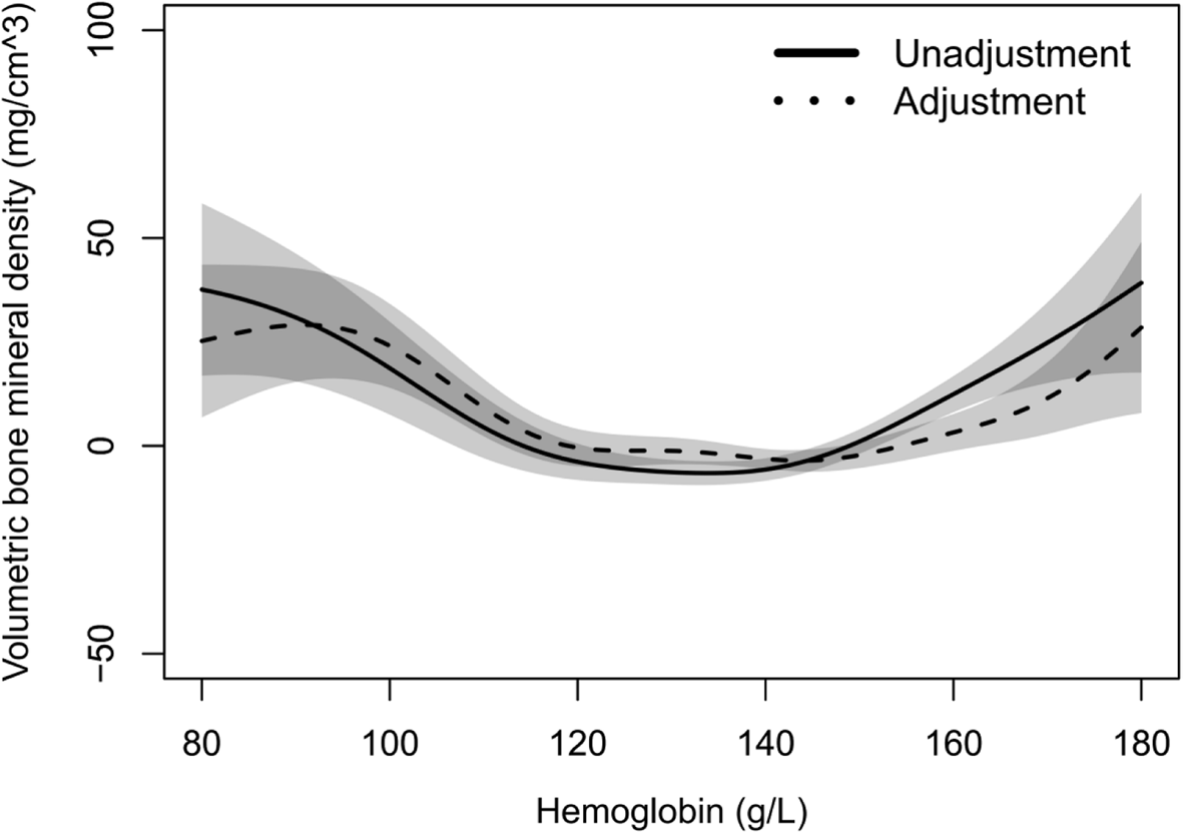
Generalized smoothing splines for vBMD and HGB. Horizontal coordinates represent different components, while vertical coordinates represent residuals of vBMD. Solid line: no adjustment; dashed line: adjusted for gander, age, BMI, TC, TG, SBP, DBP, GLU, HDL and LDL. Shaded area shows the 95% confidence interval

Figure 1 shows a U-shaped curve relationship between vBMD and HGB, the cut off value of HGB was 130 g/L. vBMD decreased with HGB when HGB < 130 g/L, and increased when HGB ≥ 130 g/L, before and after adjustment for gender, age, BMI, TC, TG, SBP, DBP, GLU, HDL and LDL. These results are presented on Table 2.

**Table 2:** Association between vBMD and HGB by linear regression model

HGB (g/L)	Unadjusted				HGB (g/L)	Adjusted^a^			
	B	S.E	*t*	*P*		B	S.E	*t*	*P*
80 ≤ HGB < 130	-0.81	0.26	-3.12	0.002	80 ≤ HGB < 130	-0.57	0.17	-3.44	0.001
130 ≤ HGB < 180	0.81	0.12	6.59	0.000	130 ≤ HGB < 180	0.41	0.12	3.36	0.001

^a^ Adjusted for gender, age, BMI, TC, TG, SBP, DBP, GLU, HDL and LDL

### Relationship between vBMD and HGB in different genders

**Fig. 2:**
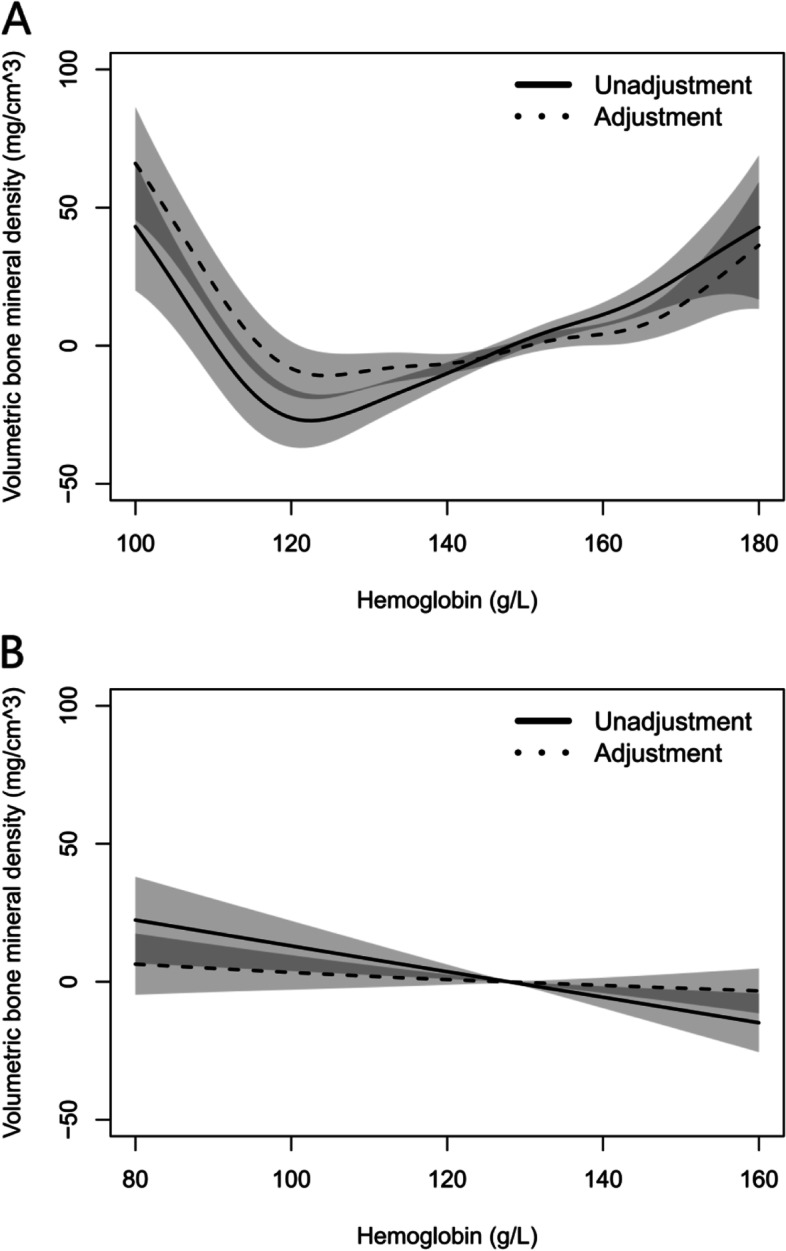
Generalized smoothing splines for vBMD and HGB in male group (**A**) and female group (**B**). Horizontal coordinates represent different components, while vertical coordinates represent residuals of vBMD. Solid line: no adjustment; dashed line: adjusted for age, BMI, TC, TG, SBP, DBP, GLU, HDL and LDL. Shaded area shows the 95% confidence interval

Figure 2 shows that through gender stratification and before and after adjustment for age, BMI, TC, TG, SBP, DBP, GLU, HDL and LDL, a U-shaped curve relationship was established between vBMD and HGB in male group, vBMD decreased with HGB when HGB < 120 g/L, and increased when HBG ≥ 120 g/L; while a negative linear relationship between vBMD and HGB in female group. These data are shown on Table 3.

**Table 3:** Association between vBMD and HGB by linear regression model in different genders

Gender	HGB (g/L)	Unadjusted				HGB (g/L)	Adjusted^a^			
		B	S.E	*t*	*P*		B	S.E	*t*	*P*
Male	100 ≤ HGB < 120	-3.95	0.61	-6.46	0.000	100 ≤ HGB < 120	-3.89	0.65	-6.01	0.000
	120 ≤ HGB < 180	1.08	0.13	8.66	0.000	120 ≤ HGB < 180	0.55	0.12	4.71	0.000
Female	80 ≤ HGB < 160	-0.47	0.16	-2.86	0.004	80 ≤ HGB < 160	-0.11	0.11	-0.99	0.321

^a^Adjusted for age, BMI, TC, TG, SBP, DBP, GLU, HDL and LDL

## Discussion

A statistical survey has shown that the prevalence of osteoporotic fractures in women over 50 years old in China was 15%, with the prevalence of osteoporosis being significantly high among people over 60 years old, especially among female patients [29, 31, 32]. In recent years, epidemiological and clinical studies have found that HGB levels are associated with osteoporosis [33, 34]. It suggested that low oxygen environment may affect bone metabolism, while anemia may cause bone loss, reduce vBMD, and lead to osteoporosis [23].

The results of this study are not consistent with other studies. In this study, after adjusting the age and others factors, the relationship between vBMD and HGB in male population is presented a U-shaped curve, when HGB < 120 g/L, the vBMD decreases with HGB increasing, while when HGB ≥ 120 g/L, the vBMD increases with HGB increasing. The possible reason is that higher HGB level means that the body has a strong ability to provide oxygen feedback, indicating that the exercise amount of these people in daily life is higher, and the exercise is positively related to bone density. In clinical, low level of HGB is an important index of iron deficiency anemia when HGB was lower than 120 g/L [34]. There is a mechanism to promote bone formation in clinic, that is, iron deficiency anemia caused by blood loss can promote the proliferation of bone cells [35]. This negative feedback regulation may cause a negative correlation between vBMD and HGB at low HGB level (HGB < 120 g/L). Meanwhile, the HGB level is closely related to the content of red blood cells, and the production of red blood cells depends on the content of red marrow, with the increase of age, red marrow gradually changes to yellow marrow, and the decrease of red marrow leads to the decrease of RBC productivity, which finally results in the decrease of HGB. At the same time, with the increase of age, bone density level will also decrease [35]. Consistent with the results of this study is that Kri and Kim showed the positive correlation between vBMD and HGB in male population [24, 25]. And, different from the linear regression analysis method used in other studies, the smooth curve used in this study can further explore the quantitative relationship between vBMD and HGB. Furthe more, the subjects in these studies was only for the elderly (age > 50), and this study included a wider age group (age 20–85), so it can better show the role of age in the relationship between them. To further assess whether the age inclusion criteria are the reason why our results differ from other studies, we conducted age stratification analysis (supplementary Fig. 1–2 and supplementary Table 1–2), the results showed that the relationship between vBMD and HGB in the two age groups in male group was still a U-curve, but the decrease of HGB < 120 g/L was relatively slow in the participants under 50 years old.

In female participants, there was no significant association of vBMD and HGB after adjusted age and other factors. It indicated that the factors corrected by this study have a great influence on the relationship between BMD and HGB. The cause maybe related to the promotion of periodic menstrual loss on osteoblast proliferation in young women. Due to this regulation, vBMD in female is maintained at a relatively stable level in young age, and it does not begin to decrease until the elderly menopause [36, 37], which suggests that age has a greater impact on vBMD for women than HGB. Similar to this study, Yun’s study also found a negative correlation between vBMD and HGB without age and other factors adjustment in female population [33].

It is worth noting that most previous studies used dualenergy X-ray absorptiometry (DXA) method to measure BMD, which can not distinguish cancellous bone from cortical bone. In fact, the area and conversion rate of cancellous bone are much higher than cortical bone [38]. In addition, in the measurement of lumbar indicators, the measured value may be higher than the actual value due to bone hyperplasia, intervertebral disc degeneration, ligament ossification and abdominal aorta calcification, which may cause misdiagnosis and missed diagnosis [39]. In contrast, the QCT method used in this study will not be interfered by factors such as bone hyperplasia, so it has a high accuracy.

In conclusion, this further suggests that gender should not be ignored when hemoglobin is used as an early warning indicator of BMD related diseases.

### Limitations of the study

There are some limitations in this study. Firstly, causal links in this study cannot be accounted by the cross-sectional study. Secondly, some important factors affecting BMD in this study were not investigated, such as menopausal or menopausal age, vitamin D intake and so on, which restricted the scientificity of the results. Thirdly, the study population is physical examination population, and its characteristics are different from the community population, so the representativeness of the research results is limited.

## Conclusions

The relationship between vBMD and HGB in male population presents a U-shaped curve. and a linear relationship in female population. At present, vBMD is an important indicator of bone strength, and it is also an important diagnostic criterion for osteoporosis. In the absence of effective treatment, active prevention of osteoporosis is very important. It is of great significance to identify some indicators for evaluating the progress of osteoporosis, and for guiding its treatment. In the future, the scope of the study will be expanded, and a multi-center research will be carried out. Further studies are required to confirm these findings and determine the potential mechanisms underlying the observed associations.

## Supplementary Information

**Additional file 1: Supplementary table 1: **Association between vBMD and HGB by linear regression model in male group. **Supplementary table 2: **Association between vBMD and HGB by linear regression model in female group. **Supplementary figure 1: **Generalized smoothing splines for vBMD and HGB in male group, 20-50 years (A), 50-90 years (B). Horizontal coordinates represent different components, while vertical coordinates represent residuals of vBMD. Solid line: no adjustment; dashed line: adjusted for age, BMI, TC, TG, SBP, DBP, GLU, HDL and LDL. Shaded area shows the 95% confidence interval. **Supplementary figure 2: **Generalized smoothing splines for vBMD and HGB in female group, 20-50 years (A), 50-90 years (B). Horizontal coordinates represent different components, while vertical coordinates represent residuals of vBMD. Solid line: no adjustment; dashed line: adjusted for age, BMI, TC, TG, SBP, DBP, GLU, HDL and LDL. Shaded area shows the 95% confidence interval.

## Data Availability

The data of participants are collected by the authors and uploaded to the database, which makes it easier for the authors to use the data in the process of analyzing data and writing manuscripts. This kind of database system can conveniently shield the data irrelevant to the experiment and effectively protect the privacy of participants. The data that support the findings of this study are available from the Health Management Center at the First Affiliated Hospital of Wannan Medical College in Wuhu, China but restrictions apply to the availability of these data, which were used under license for the current study, and so are not publicly available. Data are however available from the authors upon reasonable request and with permission of Health Management Center at the First Affiliated Hospital of Wannan Medical College in Wuhu, China. If someone wants to request the data from this study, they can contact Yufeng Wen (corresponding author).

## Abbreviations

OP: Osteoporosis
BMD: Bone mineral density
vBMD: Volumetric bone mineral density
HGB: Hemoglobin
BMI: Body mass index
SBP: Systolic blood pressure
DBP: Diastolic blood pressure
TC: Total cholesterol
TG: Triglycerides
GLU: Glucose
HDL: High-density lipoprotein
LDL: Low-density lipoprotein
WHO: World Health Organization
BP: Blood pressure
QCT: Quantitative CT
