# Supplementary material for: Implications of gender-based variabilities in bone mineral density and hemoglobin levels
Source: BMC Musculoskelet Disord. 2021 Jul 30;22:645. doi: 10.1186/s12891-021-04536-7 (PMC8323312; doi:10.1186/s12891-021-04536-7)
Supplement: Supplementary file 1 — Additional file 1: Supplementary table 1: Association between vBMD and HGB by linear regression model in male group. Supplementary table 2: Association between vBMD and HGB by linear regression model in female group. Supplementary figure 1: Generalized smoothing splines for vBMD and HGB in male group, 20-50 years (A), 50-90 years (B). Horizontal coordinates represent different components, while vertical coordinates represent residuals of vBMD. Solid line: no adjustment; dashed line: adjusted for age, BMI, TC, TG, SBP, DBP, GLU, HDL and LDL. Shaded area shows the 95% confidence interval. Supplementary figure 2: Generalized smoothing splines for vBMD and HGB in female group, 20-50 years (A), 50-90 years (B). Horizontal coordinates represent different components, while vertical coordinates represent residuals of vBMD. Solid line: no adjustment; dashed line: adjusted for age, BMI, TC, TG, SBP, DBP, GLU, HDL and LDL. Shaded area shows the 95% confidence interval. [file 12891_2021_4536_MOESM1_ESM.docx]

**Supplementary table 1:** Association between vBMD and HGB by linear regression model in male group

| Age | HGB  (g/L) | Unadjusted | | | | HGB  (g/L) | Adjusted* | | | |
| --- | --- | --- | --- | --- | --- | --- | --- | --- | --- | --- |
|  |  | B | S.E. | *t* | *P* |  | B | S.E. | *t* | *P* |
| ≤ 50 | 120≤ HGB <130 | -1.84 | 0.71 | -2.59 | 0.027 | 120≤ HGB <130 | -1.11 | 2.47 | -0.45 | 0.027 |
|  | 130≤ HGB <180 | 0.86 | 0.18 | 4.78 | 0.000 | 130≤ HGB <180 | 0.54 | 0.18 | 3.10 | 0.002 |
| > 50 | 100≤ HGB <120 | -4.91 | 0.75 | -6.53 | 0.000 | 100≤ HGB <120 | -5.33 | 1.07 | -4.99 | 0.000 |
|  | 120≤ HGB <180 | 0.73 | 0.15 | 4.93 | 0.000 | 120≤ HGB <180 | 0.53 | 0.15 | 3.46 | 0.001 |

*Adjusted for age, BMI, TC, TG, SBP, DBP, GLU, HDL and LDL.

**Supplementary table 2:** Association between vBMD and HGB by linear regression model in female group

| Age | HGB  (g/L) | Unadjusted | | | | HGB  (g/L) | Adjusted* | | | |
| --- | --- | --- | --- | --- | --- | --- | --- | --- | --- | --- |
|  |  | B | S.E. | t | P |  | B | S.E. | t | P |
| ≤ 50 | 80≤ HGB <160 | 0.07 | 0.16 | 0.41 | 0.679 | 80≤ HGB <160 | 0.13 | 0.15 | 0.85 | 0.397 |
| > 50 | 90≤ HGB <160 | -0.53 | 0.20 | -2.69 | 0.008 | 90≤ HGB <160 | -0.40 | 0.17 | -2.35 | 0.020 |

*Adjusted for age, BMI, TC, TG, SBP, DBP, GLU, HDL and LDL.


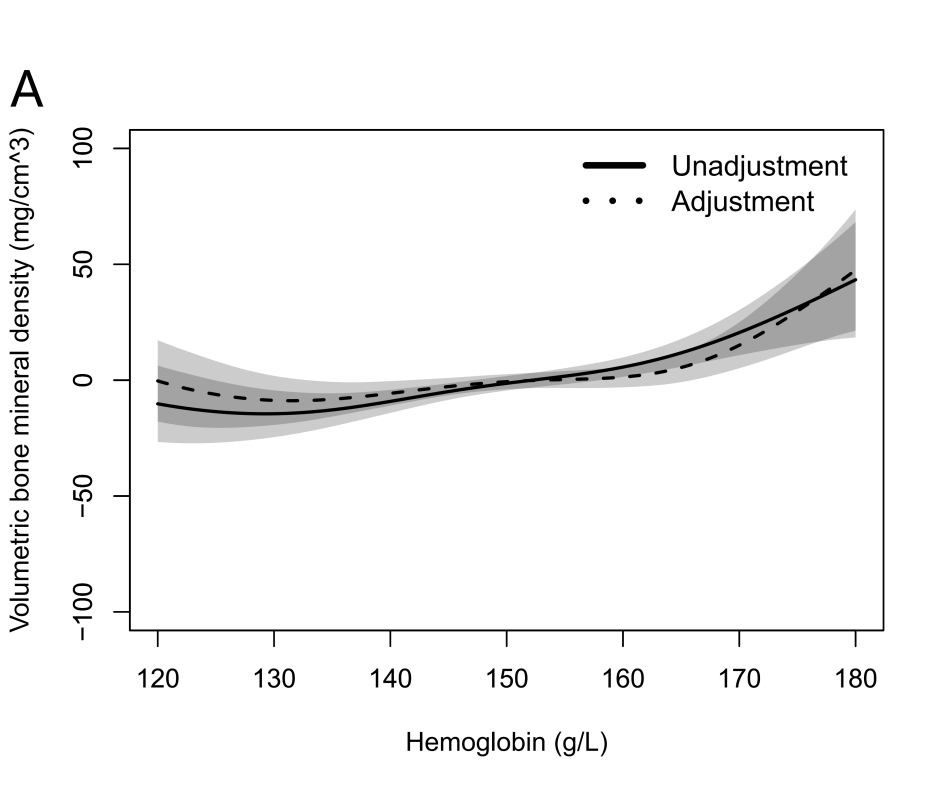

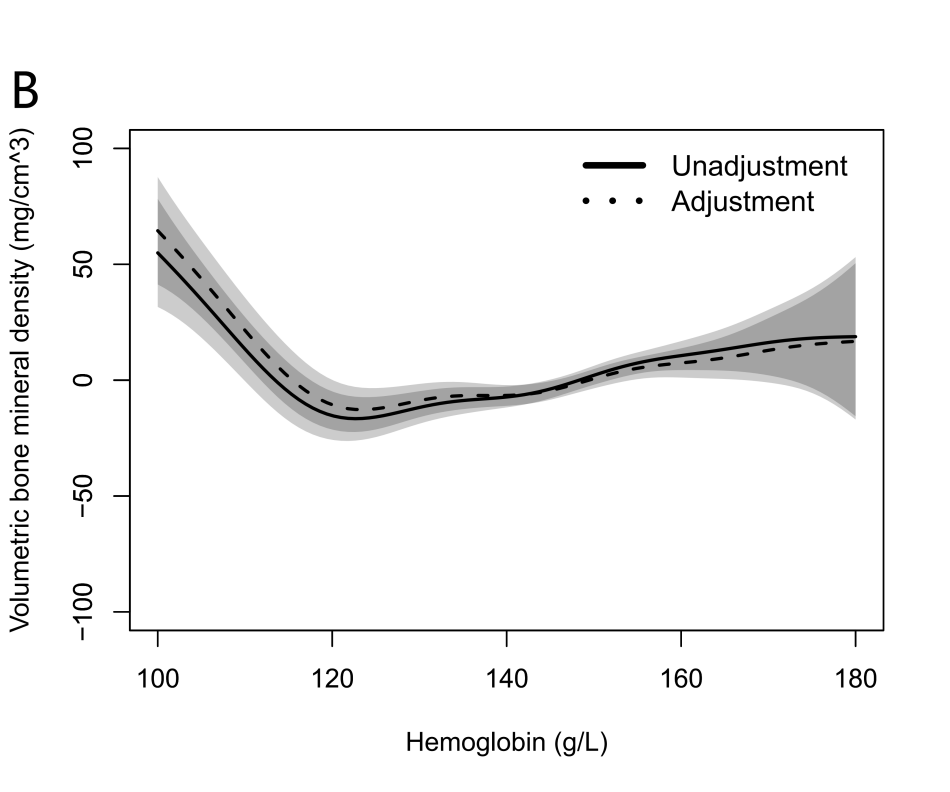


**Supplementary figure 1:** Generalized smoothing splines for vBMD and HGB in male group, 20-50 years (A), 50-90 years (B).

Horizontal coordinates represent different components, while vertical coordinates represent residuals of vBMD. Solid line: no adjustment; dashed line: adjusted for age, BMI, TC, TG, SBP, DBP, GLU, HDL and LDL. Shaded area shows the 95% confidence interval.


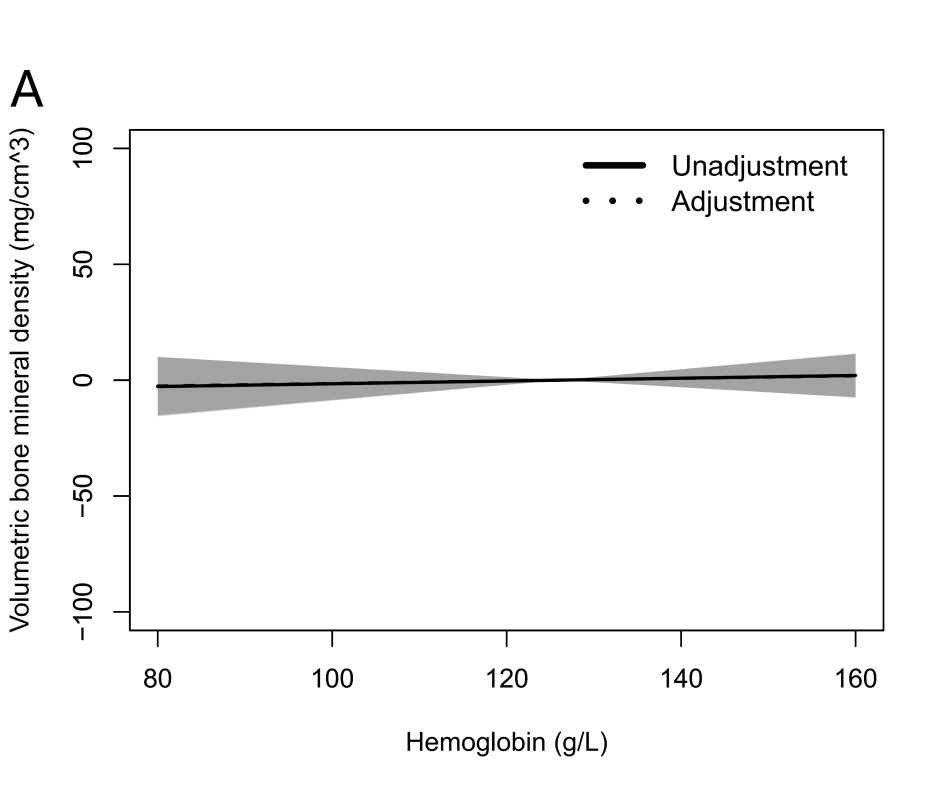

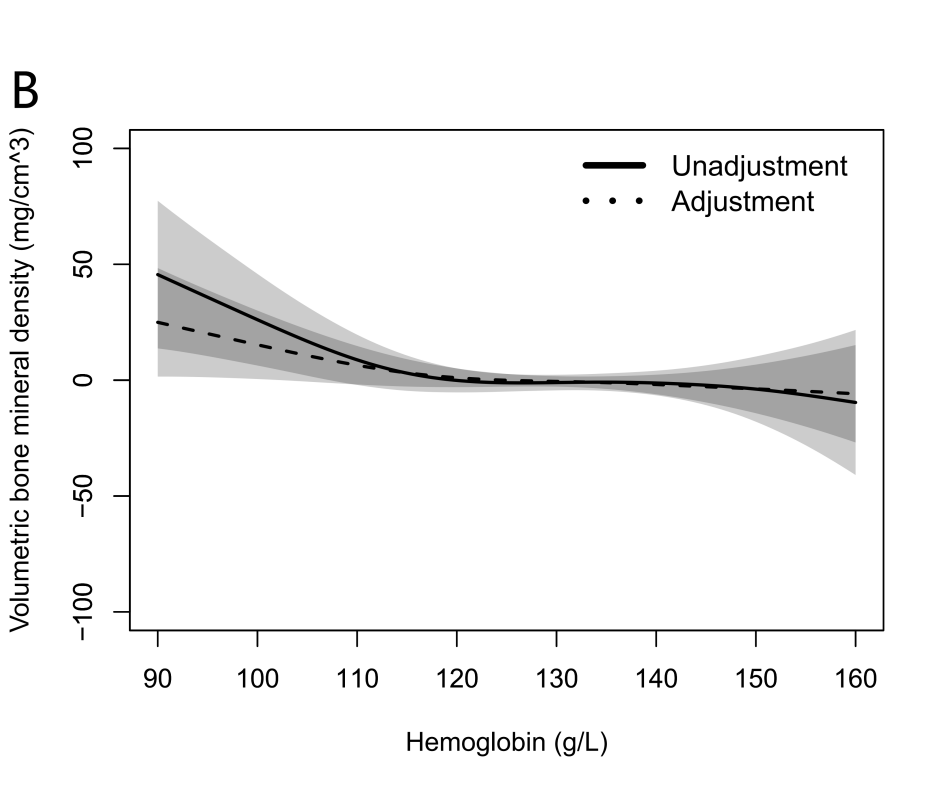


**Supplementary figure 2:** Generalized smoothing splines for vBMD and HGB in female group, 20-50 years (A), 50-90 years (B).

Horizontal coordinates represent different components, while vertical coordinates represent residuals of vBMD. Solid line: no adjustment; dashed line: adjusted for age, BMI, TC, TG, SBP, DBP, GLU, HDL and LDL. Shaded area shows the 95% confidence interval.
